# Exosome-packaged miR-1246 contributes to bystander DNA damage by targeting LIG4

**DOI:** 10.1038/s41416-018-0192-9

**Published:** 2018-07-24

**Authors:** Li-Jun Mo, Man Song, Qiao-Hua Huang, Hua Guan, Xiao-Dan Liu, Da-Fei Xie, Bo Huang, Rui-Xue Huang, Ping-Kun Zhou

**Affiliations:** 1 Institute for Environmental Medicine and Radiation Health, the College of Public Health, University of South China, 421001 Hengyang, Hunan Province P.R. China; 2 Beijing Key Laboratory for Radiobiology, Department of Radiation Biology, Beijing Institute of Radiation Medicine, 100850 Beijing, P.R. China; 3 Department of Occupational and Environmental Health, Xiangya School of Public Health, Central South University, 410078 Changsha, Hunan Province P.R. China; 4 Institute for Chemical Carcinogenesis, State Key Laboratory of Respiratory, Guangzhou Medical University, 511436 Guangzhou, P.R. China

**Keywords:** DNA, Non-coding RNAs

## Abstract

**Background:**

An increasing number of studies have recently reported that microRNAs packaged in exosomes contribute to multiple biological processes such as cancer progression; however, little is known about their role in the development of radiation-induced bystander effects.

**Methods:**

The exosomes were isolated from the culture medium of BEP2D cells with or without γ-ray irradiation by ultracentrifugation. To monitor DNA damage and repair efficiency, the DNA double-strand break biomarker 53BP1 foci, comet, micronuclei, expression of DNA repair genes and NHEJ repair activity were detected. The miR-1246 targeting sequence of the DNA ligase 4 (*LIG4*) mRNA 3′UTR was assessed by luciferase reporter vectors.

**Results:**

miR-1246 was increased in exosomes secreted from 2 Gy-irradiated BEP2D cells and inhibited the proliferation of nonirradiated cells. The miR-1246 mimic, exosomes from irradiated cells, and radiation-conditioned cell culture medium increased the yields of 53BP1 foci, comet tail and micronuclei in nonirradiated cells, and decreased NHEJ efficiency. miR-1246 downregulated LIG4 expression by directly targeting its 3′UTR.

**Conclusions:**

Our findings demonstrate that miR-1246 packaged in exosomes could act as a transfer messenger and contribute to DNA damage by directly repressing the *LIG4* gene. Exosomal miR-1246 may be a critical predictor of and player in radiation-induced bystander DNA damage.

## Introduction

Ionising radiation (IR), a double-edged sword used in diagnostic and medical therapeutic implements, is also known to cause damage to normal tissue and cellular genomic instability, i.e., chromosomal aberrations.^1^ It is becoming increasingly acknowledged that IR exposure produces radiation-induced bystander effects (RIBEs) either in targeted cells or nonirradiated adjacent cells.^2^ RIBEs have critical applications or considerations in the fields of human cancer radiotherapy, personalised radiotherapy, environmental radiation risk assessment, and occupational health.^3^ In vivo and in vitro studies have provided extensive evidence that, during this process, DNA damage, chromosomal aberrations, gene mutation, apoptosis, and cell death could be largely attributable to RIBEs.^4–8^ Over the past decades, an increasing number of studies have proposed that the intracellular transducers and signalling pathways and the DNA damage response (DDR) play a particularly important role in bystander effects.^9–11^ Li et al. reported that *mrt-2/hus-1/cep-1/ced-4*, four genes involved in the DDR, functioned as bystander effectors in the intra- and inter-systems of *Caenorhabditis elegans*.^9^ Siva et al. conducted a clinical trial to explore the DNA damage status in both irradiated and out-of-field nonirradiated tissues by testing a biomarker of the DDR response, γ-H2AX foci. Recently, Klammer et al. reported that existing mechanistic approaches mediating the bystander effects in the DDR may involve the following: (1) intercellular communication via gap junctions and (2) arrival of signals and factors secreted by target cells at the remote nontargeted cells by medium diffusion or via the circulation.^4^ It can be inferred that DDR bystander inducers and effectors elicited from radiation-targeted cells can be transferred with the help of certain vehicles into the abscopal normal cells, which play an important role in promoting or protecting patients undergoing radiotherapy from genomic DNA damage in normal tissues. RIBEs have been well-defined and the DDR has been described as a major determinant in the bystander effects. Questions that still remain are: What vehicles and their respective cargo can deliver the DDR bystander effects and how is this process achieved?

In recent years, exosomes (30–120 nm in size, rooting in endocytic compartments) have been reported as one type of vesicle released by various cell types into the extracellular environment.^12–15^ The emerging role of exosomes as vehicles has drawn great attention since they have been particularly associated with cellular communication.^16^ To serve as a vehicle, exosomes can be endocytosed by recipient cells and distinguished as a critical signalling element to mediate cellular communication.^16^ Recent reports have demonstrated that exosomes are formed and released following radiation-induced DNA damage, and p53-, DNM3-, and p65-associated pathways are activated during this process.^17–19^ These results suggest that IR can stimulate the packaging of DNA damage signalling players into exosomes. However, recent studies have also revealed that the contents of exosomes include 4563 proteins, 1639 mRNAs, and 764 microRNAs (miRNAs), indicating their typical complexity.^20–22^ Recently, studies have demonstrated that exosomes are highly useful in many beneficial or pathological physiological processes.^23,24^ Because of the multiple biological functions of exosomes, the identification of new biomolecules in exosomes and the elucidation of new mechanisms involving their communication or delivery functions are beneficial for cancer diagnosis and therapeutic applications. Here, we focus our attention on miRNAs presented in exosomes, as several previous studies, including ours, have found that miRNAs packaged in exosomes may aid in identifying the mechanism of bystander effects following IR exposure.^25–28^ miRNAs range in length from 19 to 23 nucleotides and are a class of endogenous short, noncoding RNAs that can disturb gene expression post-transcriptionally. miRNAs are considered key molecules in the regulation of protein expression and multiple cellular biological processes, including cellular growth, cell death, and differentiation.^29^ miRNAs are also associated with the DDR following exposure to IR. Chiba et al. indicated that miR-375-3p is significantly increased following exposure to 7 Gy of IR, and the authors suggested that this miRNA can serve as a predictor of DNA damage induced by IR.^30^ Interestingly, following secretion from targeted cells, miRNAs packaged in exosomes can move to a remote distance to influence cell functions and modify the niche and host reaction in targeted or nontargeted cells, which leads to the bystander effect. Our previous study indicated that miR-7-5p packaged in exosomes from 2-Gy-irradiated human bronchial epithelial BEP2D cells induced bystander autophagy in nonirradiated BEP2D cells, and this autophagy was associated with the EGFR/Akt/mTOR signalling pathway.^31^ Yin et al. used an exosome-mediated transfer model and showed that miR-21, a well-studied DDR miRNA, plays a role in RIBEs.^32^ This study also suggested that the shuttle of exosomal miRNAs plays an important role in the cellular communication between irradiated and nonirradiated cells. Therefore, miRNAs packaged in exosomes may serve as a novel mediator and regulator of RIBEs. However, details addressing the highlights of exosomal miRNA shuttle, particularly its association with the genomic instability in the RIBE process, should be clarified. Based on the evidence discussed above, we hypothesised that miR-1246, a radiation-induced miRNA, is packaged in exosomes and delivered to nonirradiated cells to cause bystander DNA damage. We investigated this hypothesis in human BEP2D and HEK-293T cells. The results of this study contribute to the discovery of the role of exosomal miRNAs in radiation-induced bystander DNA damage and uncover the signalling pathway involved.

## Materials and methods

### Cell culture and irradiation

BEP2D cells were kindly provided by Dr. C.C. Harris of the Lab of Human Carcinogenesis Division of Basic Science, National Cancer Institute, NIH, USA. HEK-293T cells were purchased from the Institute of Basic Medical Sciences, Chinese Academy of Medical Sciences (Beijing, China). BEP2D cells were cultured in serum-free LHC-8 medium (Gibco, Grand Island, NY, USA). HEK-293T cells were cultured in Dulbecco’s modified Eagle medium supplemented with 10% foetal bovine serum. The medium was supplemented with 100 U/mL penicillin and 100 µg/L gentamycin. The cells were cultured in a humidified incubator at 37 °C with 5% CO_2_. BEP2D cells were irradiated with ^60^Co γ rays at a dose rate of 1.98 Gy/min at room temperature.

### Exosome isolation

BEP2D cells were seeded onto 10-cm culture dishes (Thermo Fisher Scientific (China) Co. Ltd., Beijing, China) and incubated for 24 h. The medium was then replaced with 10 mL of fresh LHC-8 medium. Cells were irradiated with 2 Gy of ^60^Co γ rays. Following irradiation, cells were cultured for 4 or 8 h. Control cells without irradiation were used to control for experimental error. Radiation conditioned cell culture medium (RCCM) and nonirradiation control cell culture medium (CCCM) were collected from BEP2D cells. Medium was filtered through 0.2-µm filters (Pall Corporation, Beijing, China). The medium was centrifuged at 300 × *g* for 10 min, followed by 2000 × *g* for 20 min at 4 °C to remove cell debris. The supernatant was collected and centrifuged at 100,000 × *g* for 70 min, followed by 10,000 × *g* for 30 min at 4 °C. The pellets were resuspended in 100–200 µL of sterile 1× phosphate-buffered saline (PBS). Exosomal RNAs were extracted using TRIzol reagent (Sigma-Aldrich, St. Louis, MO, USA).

### Plasmids, miR-1246 mimic and inhibitor, and antibodies

EJ5-GFP plasmids were linearised by *Hin*dIII enzyme digestion and pCherry vectors were used to detect the double-strand break (DSB) repair efficiency of nonhomologous end joining (NHEJ).^33–35^ miR-1246 mimic (sense: 5′-AAUGGAUUUUUGGAGCAGG-3′, antisense: 5′-UGCUCCAAAAAUCCAUUUU-3′); miR-NC (sense: 5′-UUCUCCGAACGUGUCACGUTT-3′, antisense: 5′-ACGUGACACGUUCGGAGAATT-3′); miR-1246 inhibitor (5′-CCUGCUCCAAAAAUCCAUU-3′); and inhibitor-NC (5′-CAGUACUUUUGUGUAGUAGUACAA-3′) were purchased from GenePharma (Shanghai, China).

Antibodies used in this study were as follows: anti-53BP1 (Abcam, Cambridge, UK), anti-LIG4 (12695-1-AP; Proteintech Group Inc., Rosemont, IL, USA), anti-CD63 (H-193, sc-15363; Santa Cruz Biotechnology, Santa Cruz, CA, USA), anti-TSG101 (ab133586; Abcam, Cambridge, MA, USA), anti-GAPDH (TA309157; Beijing Zhongshan Jinqiao Biotechnology Co., Ltd., Beijing, China), and anti-β-actin (TA-09; Beijing Zhongshan Jinqiao Biotechnology Co., Ltd.).

### Cell transfection, RNA extraction, and real-time quantitative PCR (RT-qPCR)

BEP2D and HEK-293T cells were seeded onto 60-mm plates and grown to 60% confluence, and the cells were transfected with miR-1246 mimic/control mimic or plasmids with Lipofectamine 2000 (10 or 250 µL, respectively; Invitrogen, Carlsbad, CA, USA) according to the manufacturer’s instructions. BEP2D and HEK-293T cells were harvested at the indicated timepoints after transfection and used for subsequent experiments.

Total RNA was extracted from the collected exosomes of irradiated and nonirradiated cells with TRIzol reagent (Sigma-Aldrich) according to the manufacturer’s instructions. Total RNA (1 µg) was reverse transcribed into cDNA. Mature miRNA-1246 expression was detected and quantified using the TaqMan miRNA Expression Assay Kit (Roche, Basel, Switzerland) according to the manufacturer’s instructions. U6 served as the internal control. RT-qPCR was conducted according to the fluorescent-labelled FAM Roche TaqMan Kit (Haoqin Biotech, Shanghai, China) on a Bio-Rad iCycler and iQ Real-Time PCR system (Bio-Rad, Hercules, CA, USA). For statistical analysis, each sample was repeated three times and three independent experiments were performed. miR-1246 expression in the blank control cell group was used to determine the relative expression level in irradiated cells.

BEP2D cells were harvested 24 and 48 h after transfection with the miR-1246 mimic or the miR-negative control (NC). Total RNA was extracted using TRIzol reagent. Total RNA (1 µg) was reverse transcribed into cDNA using ReverTra Ace (Toyobo, Osaka, Japan). RT-qPCR was performed to detect *LIG4*, *GTF2H5*, *ERCC4*, and *RAD51AP1* expression levels using a Bio-Rad iCycler and iQ Real-Time PCR system (Bio-Rad) with a fluorescence-labelled SYBR Green Real-Time Master Mix Kit (TIANGEN Biotech (Beijing) Co., Ltd., Beijing, China). β-actin was used as an endogenous control. The sequences of the forward and reverse primers for these genes and β-actin were as follows:

*LIG4*, forward 5′-AGCAAAAGTGGCTTATACGGATG-3′ and reverse 5′-TGAGTCCTACAGAAGGATCATGC-3′; GTF2H5, forward 5′-AAGACATTGATGACACTCACGTC-3′ and reverse 5′-GGGAAAAAGCATTTTGGTCCATT-3′; ERCC4, forward 5′-GGAACTGCTCGACACTGACG-3′ and reverse 5′-GCGAGGGAGGTGTTCAACTC-3′; RAD51AP1, forward 5′-ATGACAAGCTCTACCAGAGAGAC-3′ and reverse 5′-CACATTAGTGGTGACTGTTGGAA-3′; and β-actin, forward 5′-ATCACCATTGGCAATGAGAG-3′ and reverse 5′-TTGAAGGTAGTTTCGTGGAT-3′. Each sample was repeated three times, and the expression of *LIG4*, *GTF2H5*,* ERCC4*, and *RAD51AP1* in the miR-NC group was used to determine the relative expression level in the treated cells.

### Cell proliferation assay

The cell counting kit-8 (CCK-8) colorimetric assay (DOJINDO Molecular Technologies, Inc., Kumamoto, Japan) was used to assess cell proliferation. To produce the orange coloured product, the WST-8 agent, 2-(2-methoxy-4-nitrophenyl)-3-(4-nitrophenyl)-5-(2,4-disulfophenyl)-2H-tetrazolium, monosodium salt was added to the cell culture medium. The amount of formazan dye generated by dehydrogenases in cells is directly proportional to the number of living cells. BPE2D cells were transfected with 50 nM of the miR-1246 mimic or miR-NC. After 4 h, the transfected cells were plated in 96-well plates at a density of 5×10^3^ cells/well and cultured at 37 °C in 5% CO_2_ for the indicated times. Each sample was assayed in triplicate. Cell viability was determined at 24, 48, and 72 h using the CCK-8 assay. The optical density (OD) of each well was read on a Multiskan GO microplate reader (Thermo Fisher Scientific, Waltham, MA, USA) at 450 nm to determine cell viability. Each experiment was performed in triplicate.

### Comet and NHEJ repair efficiency assay

The neutral comet assay, a standard and sensitive technique to analyse DNA DSBs, was used in BEP2D cells.^36^ BEP2D cells were treated with exosomes following 2 Gy irradiation and transfected with 50 and 100 nM miR-1246 mimic or mimic-NC for 24 h, respectively. Then, cells were trypsinised and resuspended in 1× PBS to a final concentration of 1×10^4^ cells/mL. The comet assay was performed using the Comet Assay Reagent Kit for Single Cell Gel Electrophoresis (Trevigen, Gaithersburg, MD, USA) according to the manufacturer’s instructions. Cellular DNA was stained and analysed using an epifluorescence microscope at ×40 magnification (Nikon, Melville, NY, USA). The percentage of tail DNA was scored and quantified using CaspLab software. Additionally, BEP2D cells were transfected with linearised EJ5-GFP, an NHEJ reporter plasmid, and the pmCherry-N1 plasmid. pmCherry-N1 was used as a control to assess transfection efficiency. After 24 h, the treated BEP2D cells were harvested and analysed using fluorescence-activated cell sorting (FACS) to determine NHEJ repair efficiency.

### Western blot analysis

BEP2D cells were lysed in lysis buffer, subjected to sodium dodecyl sulphate-polyacrylamide gel electrophoresis, and transferred to polyvinylidene fluoride membranes. Membranes were blocked in 5% milk in Tris-buffered saline containing Tween-20 (TBST) for 1 h and incubated with the indicated primary antibody overnight at 4 °C. Membranes were then incubated with the indicated secondary antibody for 1 h and washed with TBST. The Image Quant LAS500 system was used to visualise the bands. Details of the western blot analysis can be found in our previous study.^37,38^

### Colony-forming ability

We performed a colony-forming ability assay to test the effect on BEP2D cell proliferation. Following transfection with the miR-1246 mimic, inhibitor, or NC, BEP2D cells were seeded onto 60-mm culture dishes at a density of 1000 cells/dish and cultured in a 5% CO_2_ incubator at 37 °C. After 2 weeks, the cells were stained with crystal violet. The number of microscopic colonies with more than 50 cells was counted.

### Detection of micronuclei

We assessed micronuclei in BEP2D cells using 4′,6′-diamidimo-2-phenylindole (DAPI) staining as previously described.^32^ An Olympus BX61 fluorescence microscope (Olympus, Tokyo, Japan) was used to count the number of micronuclei. Giemsa staining was also used to detect micronuclei in AHH-1 cells as previously described.^39^

### Dual luciferase reporter assay

Wild-type* LIG4* mRNA 3′-untranslated region (3′UTR) and mutant sequences at the predicted target sites for miR-1246 in the *LIG4* mRNA 3′UTR were cloned into the pmirGLO vector to generate the pmirGLO-LIG4_3′UTR_wt and pmirGLO-LIG4_3′UTR-Mut constructs, respectively. Cells were seeded onto 24-well plates (6.0×10^4^ cells/well) for approximately 24 h before cotransfection with 1 μg of the reporter plasmid and 1 μg of the pmirGLO internal control plasmid. After 8 h, the medium was replaced and cells were transfected with 100 nM miR-1246 mimic or control NC. After incubation for 48 h, the transfected cells were lysed and luciferase activity was detected using a Dual-Luciferase Reporter Assay System (Cat. No. E1910; Promega, Madison, WI, USA). Firefly luciferase activity was normalised to that of Renilla luciferase and each group was assayed in triplicate.

### Immunofluorescence staining laser confocal assay

DNA DSBs were analysed by quantifying the amount of dissolution of DSB biomarker 53BP1 foci by immunofluorescence staining as previously described.^40^ BPE2D and HEK-293T cells were cultured in glass chamber slides and treated with 5, 25, and 50 nM miR-1246 mimic or miR-NC, fixed with 4% paraformaldehyde, permeabilised with 0.5% Triton X-100, immunostained with the 53BP1 primary antibody, and incubated with the Alexa Fluor 488 goat anti-rabbit secondary antibody. Cells were incubated for the indicated times. Nuclei were visualised following fluorescent staining with DAPI. The number of 53BP1 foci per nucleus was counted.

### Statistical analysis

All data are presented as the mean ± standard deviation. Statistical analysis was conducted using the Student’s *t* test. *p* *<* 0.05 was considered to indicate statistical significance. SPSS 13.0 software (IBM, Armonk, NY, USA) was used for all statistical analyses.

## Results

### Radiation-inducible BEP2D-secreted exosomal miRNA-1246 regulates cell proliferation and colony formation

**Fig. 1:**
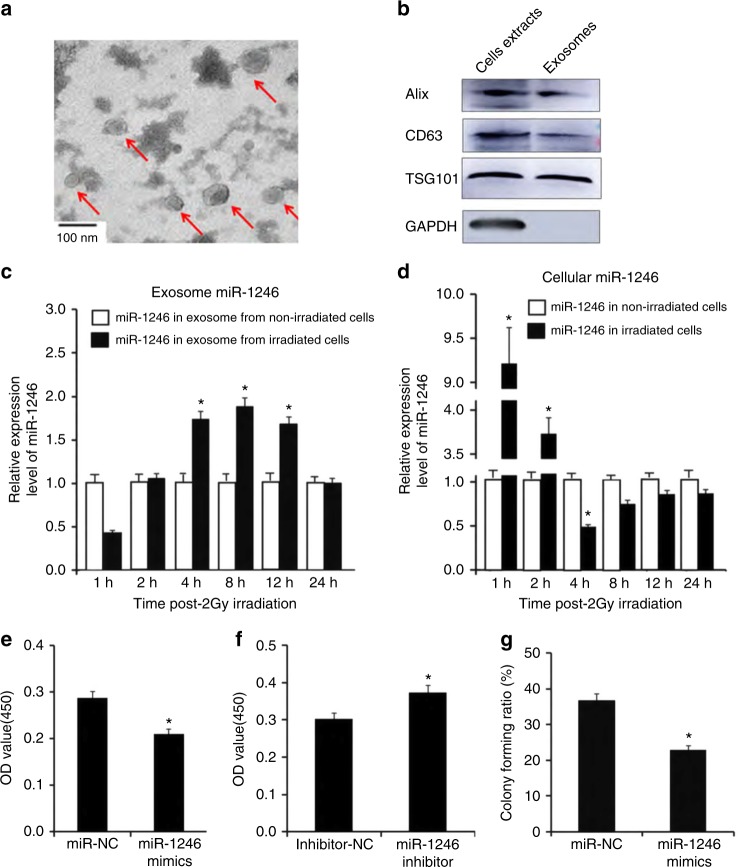
Increased expression of miR-1246 in exosomes secreted by irradiated BEP2D cells and inhibition of cell proliferation. **a** Representative image of exosomes using transmission electron microscopy. The exosomes were isolated from the culture medium of 2 Gy-irradiated BEP2D cells collected at 4 h postirradiation. **b** Western blot analysis of exosomal marker proteins. **c** Changes in expression of miR-1246 in exosomes of γ-ray-irradiated BEP2D cells. Exosomes were isolated from the culture medium of 2 Gy-irradiated BEP2D cells or control nonirradiated cells at 1−24 h postirradiation. miRNA expression levels in the exosomes were detected by qPCR. miR-1246 expression data were normalised using miR-16 as an internal control as described previously.^54^ #*p* *<* 0.01 as compared with the expression of miR-1246 in exosomes from nonirradiated control cells at the same timepoint. **d** Expression of intracellular miR-1246 was detected in BEP2D cells at 1 and 24 h post 2 Gy irradiation by qPCR. The RNA U6 was used as an internal control for intracellular miR-1246. #*p* *<* 0.01 as compared with the expression of miR-1246 in nonirradiated control cells. **e** Inhibition of miRNA-1246 mimic on BEP2D cell proliferation. BEP2D cells were transfected with 50 nM miRNA-1246 mimic or miR-NC and cell proliferation was evaluated using the CCK-8 assay at 48 h after transfection. *p *<* 0.05 as compared with control miR-NC-treated cells. **f** Enhanced proliferation of BEP2D cells by miRNA-1246 inhibitor. BEP2D cells were transfected with 50 nM miRNA-1246 inhibitor or inhibitor-NC and cell proliferation was evaluated using the CCK-8 assay at 48 h after transfection. *p *<* 0.05 as compared with control inhibitor-NC treated cells. **g** Decreased colony-forming ability of BEP2D cells by miRNA-1246 mimic. BEP2D cells were transfected with 50 nM miRNA-1246 mimic or miR-NC and colony-forming ability was assayed following miRNA transfection. *p *<* 0.05 as compared with control miR-NC-treated cells

We chose the BEP2D cell line as a model for investigating the effect of exosomes and miRNAs based on previous evidence of increased miR-1246 expression in exosomes from γ-ray-irradiated BEP2D cells by microarray analysis.^31^ To confirm that the expression level of exosomal miR-1246 changed following γ-ray irradiation and explore whether it affected cell proliferation, we isolated exosomes from culture medium of 2 Gy-irradiated BEP2D cells and nonirradiated control cells, respectively, at 4 and 8 h postirradiation by filtration and ultracentrifugation. The exosome pellets at 4 h were first examined by transmission electron microscopy as shown in Fig. 1a (8 h is shown in Supplementary Figure 1A), and further confirmed by western blot analysis of the exosome marker proteins Alix, CD63, and TSG101 (Fig. 1b). As shown in Fig. 1a, b, exosomes isolated from BEP2D conditioned medium displayed typical morphology with a size of 20–100 nm, and exosomal proteins were present in the exosomes. The sizes distribution and concentration of exosomes were also detected by nanosight analysis (Supplementary Figure 1B and C). miR-1246 was significantly increased in the exosomes of irradiated BEP2D cells compared with the control nonirradiated cells. However, the expression level of intracellular miR-1246 changed over time in irradiated cells. After 1 h of irradiation, the expression of miR-1246 was significantly increased, whereas after 4 h, the level was significantly decreased compared with control cells. When assessing the intracellular or exosomal expression level of miR-1246, we found with extension of postirradiation time that the expression level of miR-1246 in exosomes increased continuously, with a corresponding decrease in intracellular levels (Fig. 1c, d). We then investigated the effect of miR-1246 on BEP2D cell proliferation using the CCK-8 assay. Following transfection with the miR-1246 mimic, cell proliferation decreased significantly, whereas that of cells treated with the miR-1246 inhibitor increased significantly compared with miR-NC and inhibitor-NC treatment (*p* *<* 0.05) (Fig. 1e, f). Following transfection with the miR-1246 mimic, the colony-forming ratio of BEP2D cells was significantly decreased (*p* *<* 0.05) (Fig. 1g) compared with control miR-NC-treated cells. These results suggest that increased miR-1246 is specifically occurred in exosomes and mediates inhibition on cell proliferation and colony formation.

### miR-1246 can increase the yield of micronuclei in nontargeted BEP2D cells

**Fig. 2:**
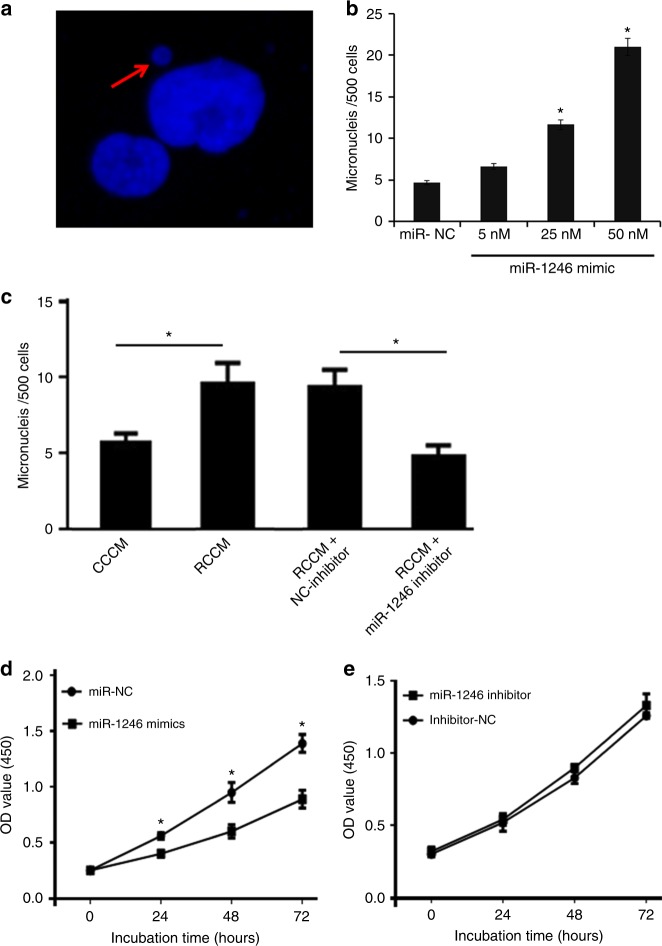
Increased yield of micronuclei in BEP2D cells transfected by miR-1246 mimics and radiation conditioned cell culture medium (RCCM). **a** Representative image of a micronucleus detected by DAPI staining. **b** Increased micronuclei in BEP2D cells following transfection with miR-1246 mimic. Micronuclei were scored in BEP2D cells following treatment with miR-1246 mimic or control miR-NC for 48 h. In total, 500 randomly selected cells were scored in each group (total number of micronuclei). *p *<* 0.01 as compared with control miR-NC cells group. **c** Induction of micronuclei in BEP2D cells by RCCM. RCCM was collected from 2 Gy γ-ray-irradiated BEP2D cell cultures at 4 h postirradiation. The nonirradiated control cell culture medium (CCCM) was used as a control. BEP2D cells were cultured in CCCM or RCCM with or without miR-1246 inhibitor or NC-inhibitor control. The micronuclei were scored 48 h later using DAPI staining. In total, 500 randomly selected cells were scored in each group (total number of micronuclei). *p *<* 0.01 as compared with miR-NC or NC-inhibitor control group. **d**, **e** Effects of miR-1246 mimic (**d**) or inhibitor (**e**) on the radiation sensitivity of BEP2D cells. In total, 5000 BEP2D cells were seeded in each well of 96-well plates. Following transfection of miR-1246 mimic, inhibitor, or NC controls for 24 h, cells were irradiated with 2 Gy of γ-rays. Proliferation was measured with the CCK-8 assay at the indicated timepoints after 2 Gy irradiation. *p *<* 0.01 as compared with the miR-NC group

To determine the role of increased miR-1246 package in exosomes in the process of RIBE, we detected the induction of micronuclei in nonirradiated BEP2D cells by miR-1246 mimic or the irradiated cells’ conditional culture medium (RCCM) with or without miR-1246 inhibitor. The cellular level of mature miR-1246 was increased significantly in BEP2D cells following the treatment of exogenous miR-1246 mimic at the concentration of 5, 25, and 50 nM compared with the miR-NC control (Supplementary Figure 2). Increased micronuclei in BEP2D cells were present following treatment with 5, 25, and 50 nM miR-1246 mimic (Fig. 2). In Fig. 2a, the red arrow indicates a representative micronucleus. Figure 2b shows that the number of micronuclei/500 cells increased significantly following treatment with 5, 25, and 50 nM miR-1246 mimic compared with the control miR-NC cells. Furthermore, both the irradiated cells’ cultures (RCCM) and nonirradiated cells’ cultures (CCCM) were collected to determine the induction of micronuclei. Figure 2c shows that the number of micronuclei/500 cells increased significantly in BEP2D cells cultured in RCCM compared with CCCM, and decreased significantly in RCCM plus miR-1246 inhibitor compared with RCCM plus NC-inhibitor. The increased induction of micronuclei was also observed in normal human lymphoblastoid AHH-1 cells by miR-1246 mimic or RCCM (Supplementary Figure 3A-C). Figure 2d shows that the OD value of cells treated with miR-1246 mimic decreased significantly compared with NC controls, although it slightly increased following incubation with miR-1246 inhibitor (Fig. 2e). Our previous study identified miR-1246 as an irradiation-inducible miRNA in exosomes from irradiated BEP2D cells.^31^ These results suggest that miR-1246 could be packaged by exosomes and transferred from targeted cells to nontargeted cells, resulting in bystander effects.

### Induction of bystander DNA damage by exosome-packaged miR-1246 in BEP2D cells by exosomes, RCCM, and miR-1246

**Fig. 3:**
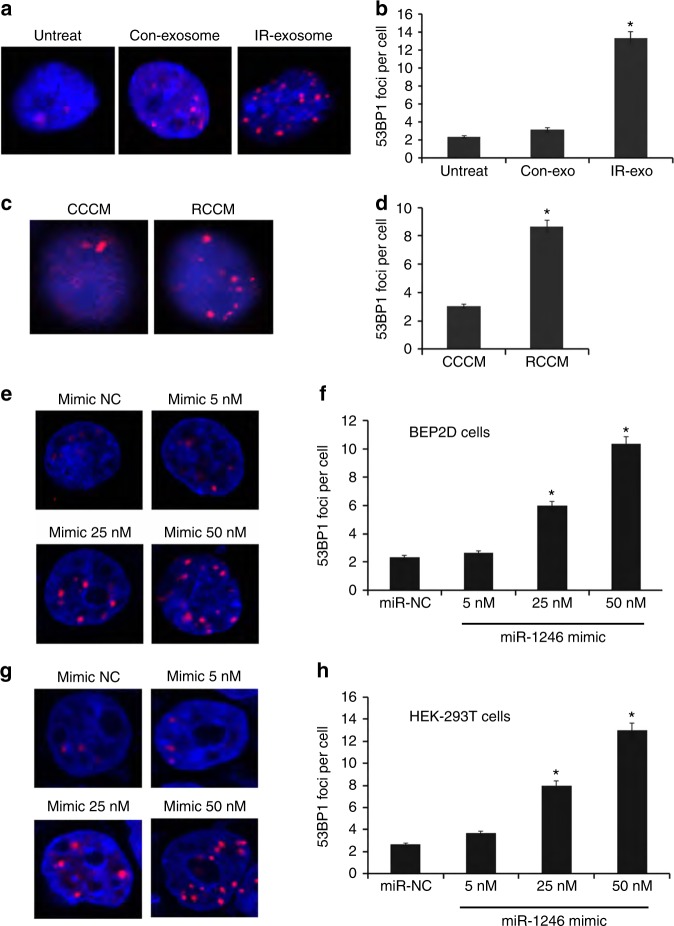
Increased induction of 53BP1 foci of DNA double-strand breaks (DSBs) in BEP2D cells by exosomes, RCCM, and miR-1246. **a** 53BP1 foci of DSBs in BEP2D cells induced by IR-exosomes from 2 Gy-irradiated BEP2D cells isolated at 4 h postirradiation. Exosomes were isolated from culture medium (3.8×10^9^ particles per 10 mL) (Supplementary 1B and C). **b** Quantitative determination of 53BP1 foci in BEP2D cells following treatment of IR-exosomes for 24 h with or without miR-1246 inhibitor. In total, 50 randomly selected cells were scored in each group (mean ± standard deviation (SD)). *p *<* 0.01 as compared with con-exosomes isolated from nonirradiated BEP2D cells. ^#^*p* *<* 0.01 as compared with IR-exosomes. **c** 53BP1 foci of DSBs in BEP2D cells induced by RCCM of Gy-irradiated BEP2D cells collected at 4 h postirradiation. **d** Quantitative determination of 53BP1 foci in BEP2D cells cultured in RCCM or CCCM for 24 h. In total, 50 randomly selected cells were scored in each group (mean ± SD). The exosome-free RCCM was the RCCM supernatant following the removal of exosomes by ultracentrifugation. *p *<* 0.01 as compared with the CCCM of nonirradiated control cell culture medium. ^#^*p* *<* 0.01 as compared with the RCCM group. **e** 53BP1 foci of DSBs in BEP2D cells induced by miR-1246 mimic. **f** Quantitative determination of 53BP1 foci in BEP2D cells following treatment with miR-1246 mimic for 24 h. In total, 50 randomly selected cells were scored in each group (mean ± SD). *p *<* 0.01 as compared with the control miR-NC. **g** 53BP1 foci of DSBs in HEK-293T cells induced by miR-1246 mimic. **f** Quantitative determination of 53BP1 foci in HEK-293T cells following treatment with miR-1246 mimic for 24 h. In total, 50 randomly selected cells were scored in each group (mean ± SD). *p *<* 0.01 as compared with the control miR-NC

To explore the involvement of exosomal miR-1246 in bystander effects, we isolated the exosomes secreted by IR-treated or nonirradiated BEP2D cells at 4 h postirradiation, or collected the RCCM and CCCM. We performed the quantitative determination of 53BP1 foci in the non IR-targeted BEP2D treated with exosomes from the irradiated cells or control cells (Fig. 3a, b), or cultured in the medium of RCCM or CCCM (Fig. 3c, d). As shown in Fig. 3a, b, a significantly increased yield of 53BP1 foci was demonstrated in BEP2D cells treated with the exosomes from irradiated cells (IR-exo) as compared to the cells treated with the exosomes from nonirradiated cells (Con-exo) (*p* < 0.05). Moreover, miR-1246 inhibitor largely attenuated the induction of 53BP1 foci by IR-exo. Figure 3c, d shows that the number of 53BP1 foci per cell was increased significantly in BEP2D cells cultured in RCCM compared with CCCM. Removal of the exosome from RCCM (Exo-free RCCM) decreased the yield of 53BP1 foci in nontargeted cells. The induction of 53BP1 foci was further observed in both BEP2D cells and HEK-293T cells by the treatment of miR-1246 mimic. Figure 3e–h shows that the number of 53BP1 foci increased continuously in BEP2D (Fig. 3e, f) and HEK-293T cells (Fig. 3g, h) transfected with increasing concentration of miR-1246 mimic. Overexpressed miR-1246 mimic significantly increased the induction of 53BP1 foci at 25 or 50 nM. The increased level of 53BP1 expression further suggested induction of the DDR by RCCM (Supplementary Figure 3D). These data indicated that overexpressed miR-1246 in 2 Gy-irradiated cells could be transferred to the medium and delivered to bystander cells to mediate bystander DNA damage via exosome cargo.

### Exosomal miR-1246 depresses NHEJ efficiency

**Fig. 4:**
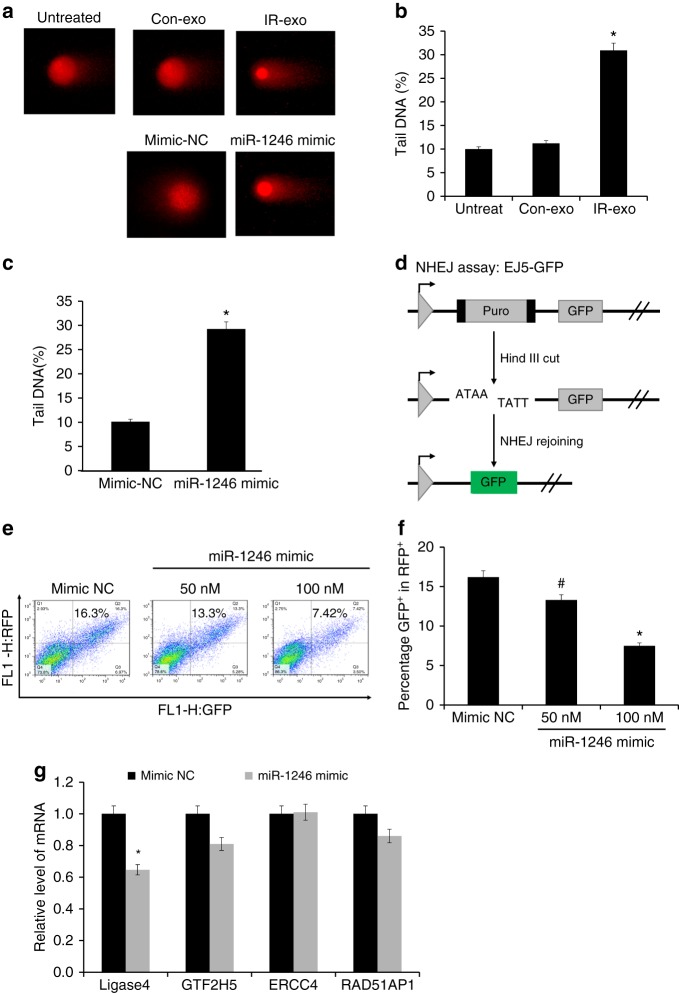
Depressed efficiency of the NHEJ pathway of DNA DSB repair by miR-1246 mimic. **a** Neutral comet assay of DNA DSBs in BEP2D cells induced by IR-exosomes or miR-1246. The comet assay was performed following treatment with IR-exosomes or miR-1246 for 24 h. **b** Percentage of tail DNA was measured and quantified using CaspLab software in cells treated with control nonirradiated cells exosomes (Con-exo) or 2 Gy-irradiated cells exosomes (IR-exo) isolated at 4 h postirradiation. The comet assay was performed in triplicate. *p *<* 0.01 as compared with the Con-exo group. **c** Percentage of tail DNA was measured and quantified using CaspLab software after 24 h treatment with control nonirradiated cell exosomes (Con-exo) or 2 Gy-irradiated cell exosomes (IR-exo) isolated at 4 h postirradiation. The comet assay was performed in triplicate. *p *<* 0.01 as compared with the Con-exo group. **d** Percentage of tail DNA was measured and quantified using CaspLab software following 24 h treatment with miR-1246 mimic or mimic-NC. The comet assay was performed in triplicate. *p *<* 0.01 as compared with the mimic-NC group. **d** Diagram of the NHEJ reporter assay. EJ5-GFP contains a promoter separated from a GFP coding cassette by a puro gene flanked by two *Hin*dIII sites. Prior to transfection, the EJ5-GFP plasmids were linearised by *Hin*dIII digestion. The promoter and GFP sequence can be rejoined by NHEJ repair. **e** Fluorescence-activated cell sorting (FACS) analysis of the effects of the miR-1246 mimic on the NHEJ efficiency of DNA DSBs. Representative FACS measurements for NHEJ pathway activity. BEP2D cells were transfected with 1 μg of pCherry and 1 μg of linearised NHEJ-GFP reporter plasmid. The pCherry plasmids express red fluorescence protein (RFP). **f** Quantification of the NHEJ assay. Relative NHEJ efficiency was measured and expressed as the percentage of GFP^+^ cells among pCherry^+^ cells. ^#^*p* *<* 0.05, *p *<* 0.01 as compared with the miR-NC control group. GFP-positive (GFP^+^) cells represent the linearised NHEJ-GFP plasmids joined by the NHEJ pathway of DSB repair. **g** Effect of miRNA-1246 mimic on the mRNA expression of a set of DNA repair genes, detected by qPCR. Decreased expression of ligase IV (*LIG4*) mRNA by miRNA-1246 mimic was detected

To further verify that exosomal miR-1246 plays a critical role in the bystander DNA damage effect, we performed neutral comet and NHEJ assays. Our neutral comet assay confirmed the induction of DNA damage by the exosomes isolated from RCCM of irradiated cells (IR-exo) or miR-1246 mimic. As shown in Fig. 4a, b, IR-exo treatment significantly increased the formation of a fluorescence-tailing phenomenon as compared to untreated control or the treatment of Con-exo, which indicated the fragmented DNA had moved forward in the gel as the consequence DNA damage (Fig. 4a, b). Cells transfected with 50 nM miR-1246 mimic showed also a significantly increased tail DNA (%) compared with that of mimic-NC (Fig. 4a, c). NHEJ is the major repair mechanism in mammalian cells. The EJ5-GFP reporter system is often used to detect NHEJ efficiency. Here, we measured NHEJ activity in BEP2D cells transfected with different concentration of miR-1246 mimic (0, 50, and 100 nM) by transfecting with 1 μg of pCherry and 1 μg of linearised NHEJ-GFP reporter plasmids, of which the pCherry plasmid expresses the red fluorescence protein (RFP^+^). NHEJ efficiency is expressed as percentage GFP-positive (GFP^+^) in the pCherry^+^ cells (RFP^+^) detected by FACS. The result indicated that at 50 nM of miR-1246 mimic, the percentage of GFP^+^ in RFP^+^ cells was 13.3%, whereas at 100 nM, the percentage was 7.42%. A significant bystander effect of decreased NHEJ efficiency was present in cells transfected with miR-1246 mimic (Fig. 4d–f). Using TargetScan (http:///www.targetscan.org) analysis, a number of DDR genes were predicted as potential targets of miR-2146, including *LIG4*, *GTF2H5*, *ERCC4*, and *RAD51AP1*. Therefore, we determined the effect of miR-1246 on the mRNA expression of these genes. As shown in Fig. 4c, the mRNA expression of these genes was decreased, and in particular, *LIG4* was dramatically decreased compared with the others (Fig. 4g).

### miR-1246 downregulated* LIG4* expression by directly targeting the *LIG4* 3′-UTR

**Fig. 5:**
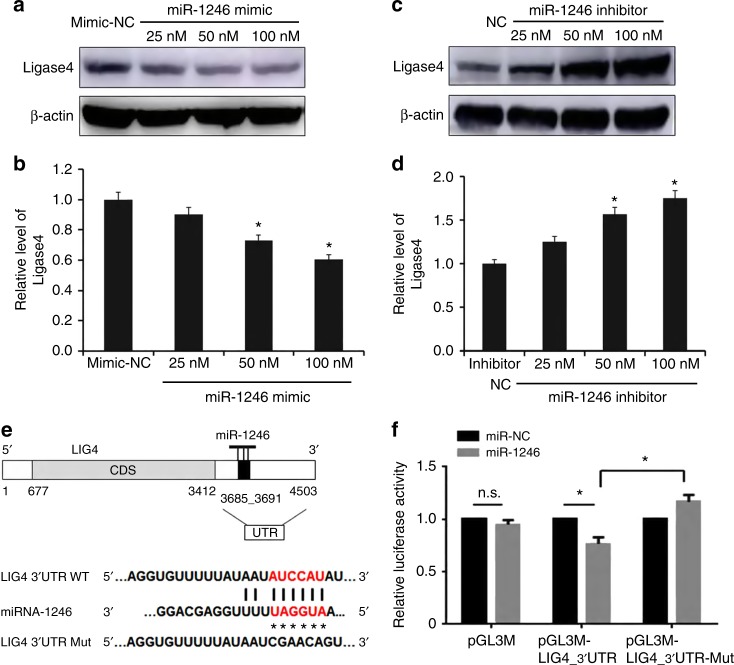
Effects of miRNA-1246 mimic on the protein expression and mRNA 3′ untranslated region (UTR) activity of the *LIG4* gene. **a** Western blot analysis of LIG4 protein expression in BEP2D cells following treatment with miR-1246 mimic for 24 h. **b** Western blot analysis of LIG4 protein expression in BEP2D cells following treatment with miR-1246 inhibitor for 24 h. **c** Densitometry of LIG4 protein expression in BEP2D cells treated with miR-1246 mimic for 24 h. Western blot analysis was performed in triplicate. *p *<* 0.01 as compared with the mimic-NC group. **d** Densitometry of LIG4 protein expression in BEP2D cells treated with miR-1246 inhibitor for 24 h. Western blot analysis was performed in triplicate. *p *<* 0.01 as compared with the inhibitor-NC group. **e** Predicted target sites of miRNA-1246 in *LIG4* 3′UTR. **f** The wild-type and mutated* LIG4* mRNA 3′UTR sequences targeted by miR-1246, which were inserted into pmirGLO plasmids to construct the luciferase reporter vectors. **g** Determination of miR-1246 interacting with the LIG4 3′UTR target sequence by detecting activity of the luciferase reporter. Cells were cotransfected with the reporter vectors and miR-1246 mimic or mimic-NC. Luciferase activity was analysed at 48 h after transfection. The reporter assay was performed in triplicate. *p *<* 0.05; n.s., not significant

To further determine whether LIG4 protein expression was altered, we performed western blot analysis and found that the protein expression of *LIG4* decreased gradually when the concentration of the miR-1246 mimic increased from 25 to 100 nM (Fig. 5a, b). Moreover, following transfection with the miR-1246 inhibitor, the LIG4 protein level tended to increase with inhibitor concentration (Fig. 5c, d). To explore the direct action of miR-1246 on the target *LIG4* mRNA 3′UTR, the pmirGLO-LIG4_3′UTR_wt and pmirGLO-LIG4_3′UTR-Mut reporter vectors were constructed. The predicted miR-1246 targeting site and sequence of the LIG4 mRNA 3′UTR are shown in Fig. 5e, f. The sequences of the 3′UTR region of *LIG4* mRNA containing the miR-1246 targeting sequence and its mutant were cloned separately in the downstream of the luciferase gene of the pmirGLO plasmid to construct the reporter vectors. Cells were then cotransfected with these reporter vectors and miR-1246 or miR-NC. miR-1246 significantly decreased the luciferase activity of the pmirGLO-LIG4_3′UTR reporter (Fig. 5g), but had no effect on the pmirGLO-LIG4_3′UTR mutant. Thus, miR-1246 can directly act on the target sequence of the *LIG4* mRNA 3′UTR to suppress LIG4 expression.

## Discussion

In 2016, our laboratory performed microarray analysis and discovered that miR-1246 was induced in secretive exosomes from 2 Gy-irradiated BEP2D cells.^31^ To further explore the biological function of miR-1246 packaged in exosomes in RIBEs, we performed a series of investigations on DNA damage effects of exosomal miR-1246, RCCM and miRNA-1246 in BEF2D cells and HEK-293T cells. Our results indicated that following irradiation, miR-1246 expression was increased in exosomes and miR-1246 mimic inhibited cell proliferation and colony formation. Moreover, miRNA-1246 in exosomes as well as in RCCM increased spontaneous DNA DSBs in BEP2D cells. These effects could be largely attenuated by treatment with miR-1246 inhibitor or removal of exosomes from RCCM. Furthermore, the NHEJ assay confirmed that miRNA-1246 decreased DNA DSB NHEJ repair efficiency. Lastly, mRNA and protein expression of *LIG4*, a critical component of the NHEJ repair pathway, were decreased significantly by exosomal miR-1246. The luciferase reporter assay strongly suggests that the* LIG4* mRNA 3′UTR is a direct target of miR-1246. These data suggest that miR-1246 packaged in exosomes in BEP2D cells plays a critical role in bystander DNA damage.

Bystander DNA damage attributed to the biological effects of IR has been well-defined in vivo and in vitro, and is generally considered as a determinant in triggering RIBEs. However, the DNA damage inducer and molecular mechanism remains unclear. Recent studies have reported that miRNAs may act as signal mediators that transmit bystander DNA damage. Tian et al. demonstrated that miR-21 plays a mediating role in bystander DNA damage, since this RNA elevates reactive oxygen species levels in nonirradiated bystander WSi cells and increases 53BP1 foci clearly.^41^ Hu et al. demonstrated that miR-663, a radiation-induced miRNA, was involved in bystander effects. It works via a feedback loop, following induction and subsequent suppression of transforming growth factor β1 expression to inhibit the transmission of the bystander signals, which are crucial in DNA damage.^42^ Previous studies indicated that miRNAs play an important role in bystander DNA damage. However, it should be mentioned that miRNAs could act as positive or negative regulators in the bystander DNA damage process depending on the context of the cells lines; therefore, the roles of miRNAs in bystander DNA damage should be studied more extensively.

We selected miR-1246 as our target miRNA because in our previous study, this miRNA was induced by IR, and another pilot study showed that this miRNA exists in exosomes.^31^ In addition, we are interested in this miRNA since emerging concern over the functions of exosomal miRNAs in RIBEs has been raised. In 2015, Xu et al. reported that miR-21 was transferred to remote nonradiated cells with the help of exosome cargo and caused bystander effects.^43^ Al-Mayah et al. used MCF7 cells to test whether bystander effects could be induced by RNA carried by exosomes. Their results suggested, at least in part, that RNA transported in exosomes plays a role in bystander effects.^44^ A study by Zhang et al. found miR-1246 could be regulated by p53 in human hepatocellular carcinoma cell lines to suppress cell proliferation and colony formation ability.^45^ Serum exosomal miR-1246 was shown to significantly inhibit prostate cancer cell growth and decrease proliferation.^46^ However, contrasting studies have also suggested different roles of exosomal miR-1246 in bystander effects. A study by Yuan et al. suggested that miR-1246 functions as an oncogene-like RNA and could act as a messenger to promote cell proliferation and enhance radiotherapy resistance between irradiation targeted and nonirradiated bystander cells by directly targeting DR5 in lung cancer cells.^47^ Similarly, breast cancer cells treated with exosomal miR-1246 could also promote cell proliferation and enhance chemotherapy resistance of nonmalignant HMLE cells.^48^ The controversial roles regarding miR-1246 might attribute to applying different tumour cell modes in those studies. Considering the phenotypes of tumour cells are usually unstable, and the function of signalling pathways is abnormal in many cases, we selected normal cell mode in our study. Consequently, results indicated that exosomal miR-1246 in the normal human bronchial epithelial BEP2D cells mediates bystander DNA damage, a key endpoint of RIBEs. It was demonstrated increased package of miR-1246 into the exosomes following radiation, and which can be transferred to adjacent nonradiated cells to induce biological functions. Considering that miR-1246 packaged in exosomes can cause DNA DSBs and suppress NHEJ efficacy, we suggest that exosomal miR-1246 can produce a detrimental effect in nonirradiated cells in the form of bystander effect. Therefore, it can be inferred that: (1) not all exosomal miRNAs induce bystander DNA damage; (2) some exosomal miRNAs could induce detrimental RIBEs, such as bystander DNA damage, whereas other exosomal miRNAs may trigger beneficial RIBEs, such as alleviating adaptive bystander DNA damage repair activity; (3) some exosomal miRNAs may serve as predictors or biomarkers to predict or test bystander DNA damage; and (4) interventions including the use of inhibitors to block specific exosomal miRNAs or washing out harmful exosomes may be employed as a radioprotection measure by blocking bystander DNA damage. However, since current studies have suggested that the functions of exosomal miR-1246 can vary, caution is needed when applying these outcomes. Further investigation regarding the molecular mechanism and the use of more extensive cell lines and animal models with outcomes obtained by standardised measurement techniques will be necessary to determine the levels of exosomal miRNAs most efficacious for various applications.

**Fig. 6:**
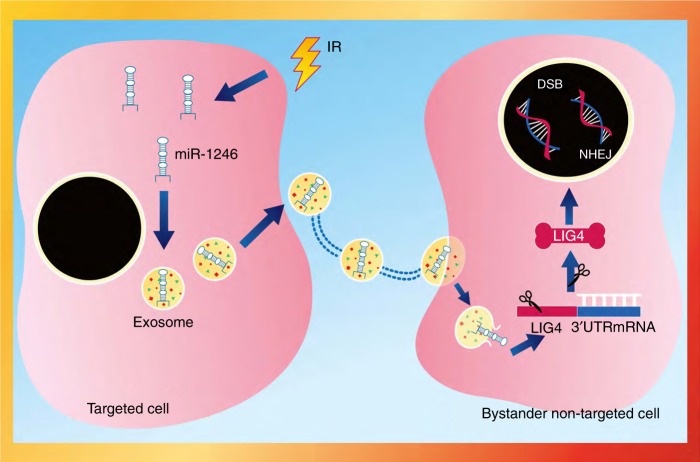
The mechanistic diagram displaying the effect of radiation-inducible exosomalmiR-1246in mediating the accumulation of DNA damage in RIBEs

*LIG4* is a key DNA repair gene in the NHEJ pathway. A number of studies have shown that *LIG4* is responsible for DSB repair and guides end-processing choice during NHEJ.^49^ Induction or overexpression of *LIG4* contributes to the radioresistance of multiple cancers.^50–52^ Our study indicated that miR-1246 suppressed *LIG4* expression, and consequently resulted in genomic DNA damage and reduced cell proliferation. The miR-1246 inhibitor reversed this effect. Based on the above evidence, we conclude that miR-1246 packaged in exosomes is transferred to nonradiated cells, in which miR-1246 induces bystander DNA damage by directly targeting *LIG4* of the DSB NHEJ repair pathway (Fig. 6). Obviously, this bystander effect is harmful to normal tissues, but may be beneficial to induce cancer cell death in radiotherapy, especially for cancer cells that overexpress LIG4. Generally, it is well recognised that IR prompts a set of miRNAs to be packaged into exosomes and, as a result, the miRNAs are transferred to adjacent or distant cells. Our study demonstrates a novel role for miR-1246 packaged in exosomes in bystander DNA damage, and our data also indicate that miR-1246 functions by directly targeting the DNA repair gene. A proposed model of miR-1246 packaged in exosomes is shown in Fig. 6 to demonstrate the role of exosomal miRNAs in affecting the maintenance of DNA damage repair machine integrity in RIBEs.

It is important to note the concerns regarding exosomal miRNAs. Which factor(s) pushes exosomes to package miRNA-1246? Are there other molecular or signalling pathways involved in the transfer process of exosomal miRNA-1246? What factor(s) releases miRNA-1246 from the cargo of exosomes after it enters bystander nontargeted cells? In bystander nontargeted cells, where miRNA-1246 binds to and inhibits the expression of *LIG4,* can we reverse this detrimental effect on DNA damage repair? These interesting questions will expand our research in the future.

However, our study has limitations that should be mentioned. First, we transfected a concentration of 25−50 nM mimic based on a previous study,^53^ which showed that this dose of mimic induced significant effects in cancer cells; however, in our study we found the effects of this dose to be modest. Thus, we chose normal cell lines instead of cancer cell lines. Second, we did not perform exosome-engineered trials to test exosome-associated miR-1246 mediation of the observed effects. It will be important to conduct these trials in future studies. Despite these limitations, our data provide evidence that miR-1246 could function as a player packaged by exosomes, and that is transferred from targeted to nontargeted cells to contribute to bystander DNA damage. Our findings could benefit further studies of exosome functions or mechanisms, as well as provide new insights into the development of radiotherapy protection or predicting the outcome of therapy.

## Electronic supplementary material

supplemental figure1

supplementary figure 2

supplementary figure 3
