# Supplementary figures and images for: Exosome-packaged miR-1246 contributes to bystander DNA damage by targeting LIG4
Source: Br J Cancer. 2018 Jul 24;119(4):492–502. doi: 10.1038/s41416-018-0192-9 (PMC6134031; doi:10.1038/s41416-018-0192-9)

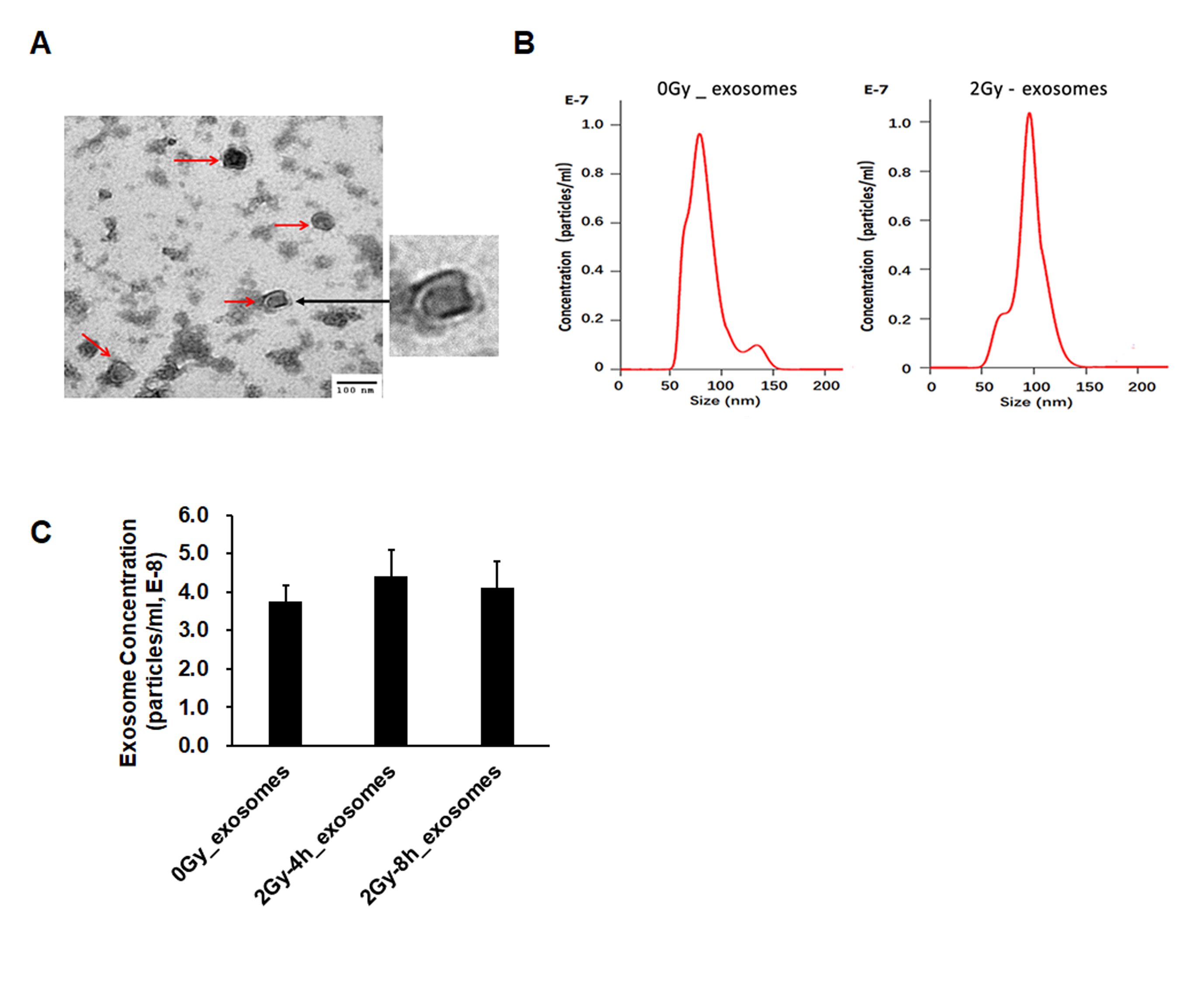

Supplement: Supplementary file 1 — supplemental figure1 [file 41416_2018_192_MOESM1_ESM.tif]

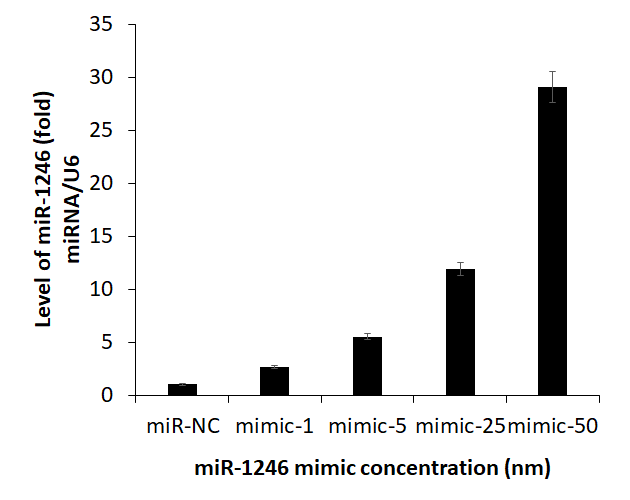

Supplement: Supplementary file 2 — supplementary figure 2 [file 41416_2018_192_MOESM2_ESM.tif]

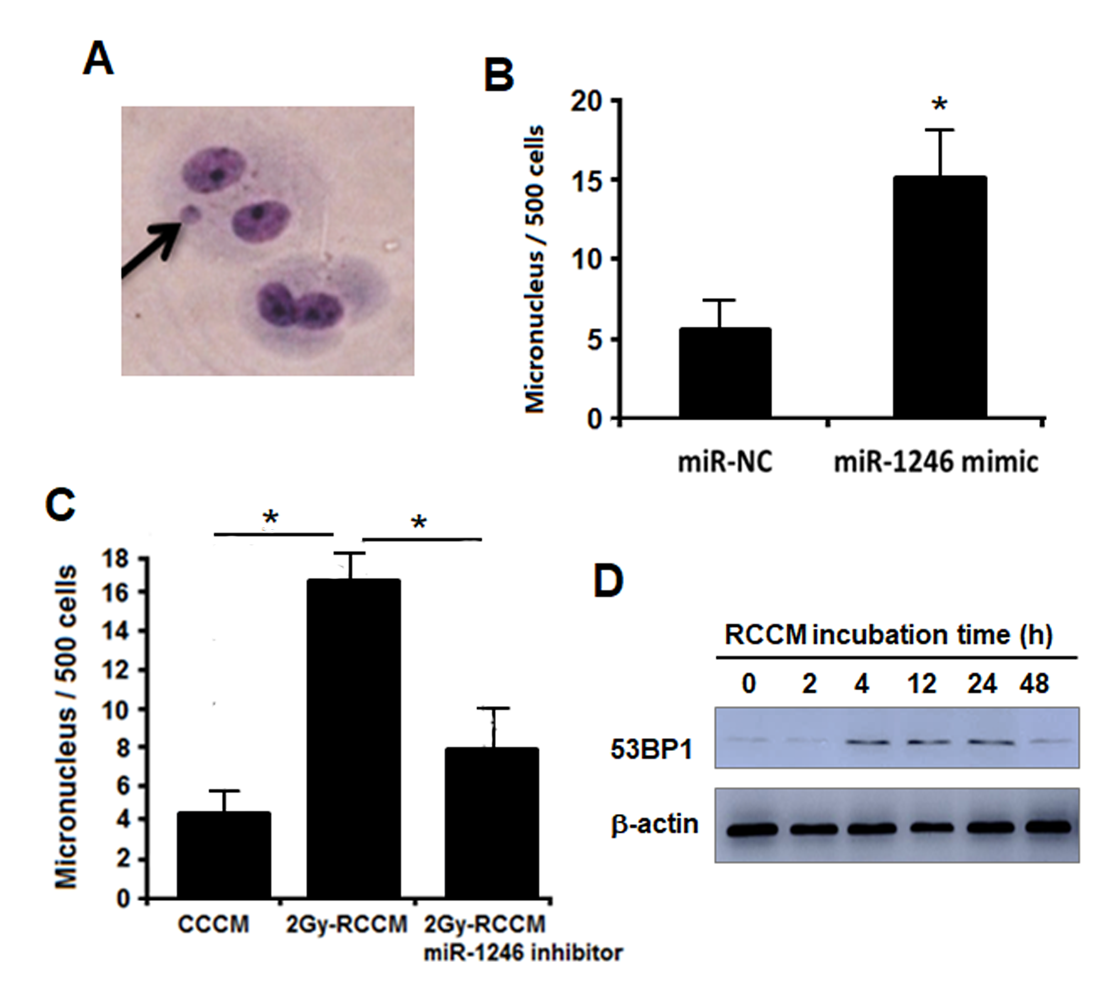

Supplement: Supplementary file 3 — supplementary figure 3 [file 41416_2018_192_MOESM3_ESM.tif]
